# Unmet Needs and Use of Assistive Products in Two Districts of Bangladesh: Findings from a Household Survey

**DOI:** 10.3390/ijerph15122901

**Published:** 2018-12-18

**Authors:** Wesley Pryor, Liem Nguyen, Qumrun Naher Islam, Faruk Ahmed Jalal, Manjula Marella

**Affiliations:** 1 Nossal Institute for Global Health, Melbourne School of Population and Global Health, The University of Melbourne, Melbourne, VIC 3000, Australia; liem.nguyen@unimelb.edu.au (L.N.); marella.m@unimelb.edu.au (M.M.); 2 Bangladesh Bureau of Statistics, Sher-e-Bangla Nagar, Dhaka 1207, Bangladesh; meghnahossain94@gmail.com; 3 Handicap International—Humanity & Inclusion, Gulshan-1 1212 Bangladesh; fa.jalal@hi.org

**Keywords:** assistive products, assistive technology, disability, LMIC, Bangladesh

## Abstract

Access to assistive products (AP) is an under-researched public health issue. Using an adaptation of a draft World Health Organization tool—the ‘Assistive Technology Assessment—Needs (ATA-N)’ for measuring unmet needs and use of AP, we aimed to understand characteristics of AP users, self-reported needs and unmet needs for AP, and current access patterns in Bangladesh. The ATA-N was incorporated in a Rapid Assessment of Disability (RAD), a population-based survey to estimate prevalence and correlates of disability. In each of two unions of Kurigram and Narsingdi districts, 60 clusters of 50 people each aged two years and older were selected using a two-staged cluster random sampling process, of whom, 4250 (59% Female; 41% Male) were adults, including 333 using AP. We estimate 7.1% of the studied population used any AP. AP use is positively associated with age and self-reported functional difficulty. The proportion of people using AP is higher for mobility than for sensory and cognitive difficulties. Of all people with any functional difficulty, 71% self-reported an unmet need for AP. Most products were home or self-made, at low cost, but provided benefits. Needs and unmet needs for AP are high, especially for people with greater functional difficulties. Assessing unmet needs for AP revealed important barriers to scale that can inform policy and practice.

## 1. Introduction

Assistive products (AP) like wheelchairs, white canes, hearing aids, communication products, visual products and many others, can transform lives by facilitating independence and participation in community life, preventing further disability, promoting early childhood development, and reducing poverty [1,2,3]. Access to an appropriate product or technology solution can be a ‘mediator or moderator’ to attaining the Sustainable Development Goals, and is therefore central to the principle of ‘leaving no-one behind’ [1]. However, the World Health Organization (WHO) estimates that worldwide, 1 billion people require AP and that the need could double by 2050 [4]. In 2018, the World Health Assembly adopted a resolution [5], which calls for greater emphasis on AP, including strengthened policy, standards, investments, and population-based data. The UN Convention on the Rights of Persons with Disabilities (CRPD), ratified by Bangladesh in 2007, and Bangladesh’s Rights and Protection of Persons with Disabilities Act (2013) compel signatories to implement effective measures for facilitating access to affordable AP and technologies.

Despite growing interest in AP in health, disability and social sectors, little is known about unmet needs and patterns of access [6]. Previous work has emphasized individual product types, rather than understanding relative access to different product categories [7,8,9] and explored AP as a mediator of rights [2], but has not explored unmet needs for AP overall. 

Previous work in Bangladesh has demonstrated that AP use is associated with greater attainment of basic rights [2], but affordability is a major barrier to AP use [6]. To inform effective strategies to scale up AP services, reliable information is needed about how people can access AP, who is at risk of missing out on the services they need, and market barriers to access. To address the lack of quality data to guide practice and policy for AP services, the WHO is developing a suite of tools, called the ‘Assistive Technology Assessment’ (ATA) comprised of three modules; i) Capacity ii) Impact, and iii) Needs. The ATA, under development by WHO, will provide a method for development and monitoring of available expertise and capacity, unmet need, and impact of AP. Using a publicly available draft ATA-Needs module in two districts of Bangladesh, we aimed to understand characteristics of AP users, self-reported needs and unmet needs for AP, and current patterns of access to AP. 

## 2. Materials and Methods 

### 2.1. Sample

This study of AP use and unmet needs was incorporated in a cross-sectional, population-based household survey undertaken in two unions of Kurigram and Narsingdi districts in Bangladesh. To determine the necessary sample size, we assumed an all-age disability prevalence of 9% [10]. This required a sample size of 2913 people per union to estimate the prevalence with a 95% confidence level, sampling error of 20%, an estimated design effect of 2.5, and a non-response rate of 20%. This required 59 clusters of 50 people each aged 2 years and above per union. The number of clusters were rounded up to 60 clusters per union.

The sampling frame comprised all enumeration areas (EAs) in the Bhogdanga Union of Kurigram Sadar Upazila in Kurigram, and the Sukundi Union of Monohordi Upazila in Narsingdi, provided by the Bangladesh Bureau of Statistics (BBS). Each EA was created to include an average of 50 households. 

Cluster random sampling involved 2 stages. In the first stage, a total of 60 EAs (clusters) out of 192 EAs from Kurigram and 60 EAs out of 80 EAs from Narsingdi were selected using a probability proportional to size procedure. Enumerators then pre-listed households within selected clusters to create a sampling frame for the second stage sampling. In the second stage, approximately 15 households (depending on the average household size) were randomly selected using a systematic sampling approach from each cluster. All eligible members of sampled households aged 2 years and older were interviewed until there were at least 50 in the cluster and all eligible members of the last household had been interviewed. When an eligible household member was absent, at least two return visits were made. Preliminary analysis compared sociodemographic characteristics for participants in the 2 districts. Subsequent analyses report pooled results of the districts unless specified otherwise. 

### 2.2. Survey Tools

The ATA-Needs tool is comprised of 4 parts (refer to Appendix A): (A) the respondent & household demographics, (B) self-reported functional difficulties (based on Washington Group Questions [11]) and self-reported needs for AP (C) AP in use, and (D) abandonment of and barriers to AP. The ATA-Needs tool was incorporated into a larger study on Rapid Assessment of Disability (RAD) surveys conducted in both districts. The survey underwent cultural adaptation through workshops with AP users, Disabled Persons’ Organization leaders, AP providers, and the Bangladesh Bureau of Statistics Health Demography unit, before translation to Bangla, backtranslation to English, and refinement of any remaining translation issues. After enumerator training, the survey was administered either directly or through a parent to each individual over 2 years old in the household to identify people at risk of disability based on activity limitations and their level of participation in the community compared to age and sex matched controls. The details of the RAD survey are reported elsewhere [12,13,14,15]. 

In this survey, the RAD demographic section and household survey were used in place of the ATA-Needs demographic section. The ATA-Needs tool was administered to adult participants (18 years and older) who identified any functional difficulties using the Washington Group Short-Set (WG) of six disability questions, as well as their age and sex matched controls without functional difficulties. Anyone who reported having at least ‘a lot of difficulty’ on one or more WG question is identified as ‘at risk of disability’. For each adult identified at risk of disability, an age (within 5 years) and sex matched control from the same cluster, who had been screened not to have disability, was identified from a neighbouring household that did not have a person with disability. To determine self-reported need for AP, the draft WHO draft tool, includes the question *‘do you think you need other assistive products that could help you with [health condition or impairment identified]’* and presenting flash-cards listing relevant assistive products based on the WHO Assistive Product Priority List. After training, surveys were administered by teams of enumerators drawn from the Bangladesh Bureau of Statistics and local disabled person’s organisations, using handheld tablets and Kobo toolkit software.

### 2.3. Data Management and Analysis

After training, surveys were administered by teams of enumerators drawn from the Bangladesh Bureau of Statistics and local disabled person’s organisations, using handheld tablets and Kobo toolkit software. Data were transferred for analysis in STATA (v15, StataCorp, College Station, TX, USA). Descriptive and bivariate analyses were used to show characteristics of AP users and non-users, the use of AP, unmet needs, facilitators and barriers for using AP. Logistic regressions were used to predict associations between various socio-economic factors with the use of AP (dichotomic variable: using vs not using AP), and with self-reported unmet need for AP among people with any functional difficulties (dichotomic variable: unmet need vs met need). Sampling weighting and adjustments to control for complex survey design effects were applied in all analyses. 

The study protocol (1647660.1) was approved by the Human Research Ethics Committee at the University of Melbourne. In Bangladesh, the study protocol and implementation were endorsed and overseen by the BBS.

## 3. Results

### 3.1. Study Population

A total of 4254 adults were surveyed (Table 1), one was excluded during cleaning due to data corruption. Using the WG short-set of six questions, 3.9% of adults (4.7% in Kurigram and 3.2% in Narsingdi) were persons with disabilities, while 31.9% reported experiencing ‘some’ functional difficulty.

The mean age of the study population was 37.9 (95% CI: 37.3–38.5) years. As expected, the presence of disability was associated with increasing age, and older people were more likely to report functional difficulties. Two-thirds (64.7%) of the population in the study areas had ever attended school. School attendance was significantly higher for people without disabilities than people with disabilities and reduced significantly with increasing level of functional difficulties; 73.9%, 45.7%, 36.6% and 29.7% of the population reporting no difficulty, some difficulty, a lot of difficulty and cannot do at all respectively, had ever attended school. About one-third (33.8%) of the study population were economically active; this proportion was highest among the population without some form of disability (35.7%), and lower for the population reporting some difficulty (30.9%), a lot of difficulty (23.9%), and cannot do at all (10.6%) respectively. 

### 3.2. The Use of Assistive Products

Overall, 7.1% of the studied population, regardless of their functional difficulty status, used AP. This included those who reported using glasses but no functional difficulty in the Washington Group questions. Table 2 summarizes factors associated with AP use. Use of AP increased with age with 18.4% of people over 60 years using AP, while 2.7% of 18–24 year olds used AP. Use of AP also increased with level of functional difficulties. Nearly two-fifths (39.5%) of the sample reporting ‘cannot do at all’ on at least one domain used AP. While Kurigram has a higher disability prevalence than Narsingdi district, prevalence of AP usage in Kurigram is lower: 5.7% compared to 8.6% in Narsingdi.

Table 2 presents odds ratios from multivariate analysis using logistic regression predicting the use of APs. After controlling for all other covariates presented in this table, having a higher level of difficulty, being older, having attended school, and living in Narsingdi were associated with higher likelihood of using APs.

**Figure 1:**
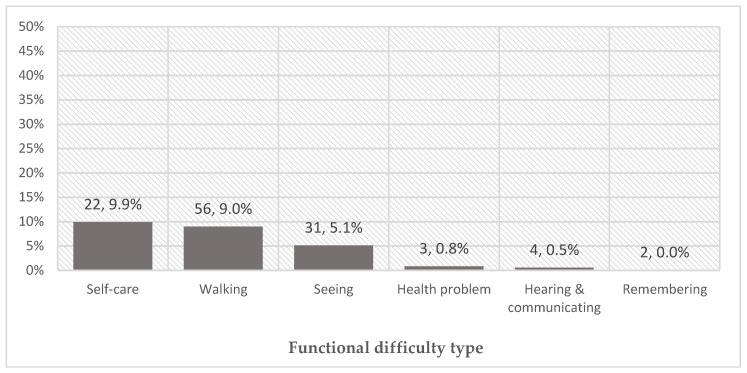
Proportion of population using assistive products (AP) among those who self-reported at least some functional difficulty (number using product, % of people reporting using AP to mitigate a difficulty in that domain); reflects use of products in more than one domain (*n* = 6).

**Figure 2:**
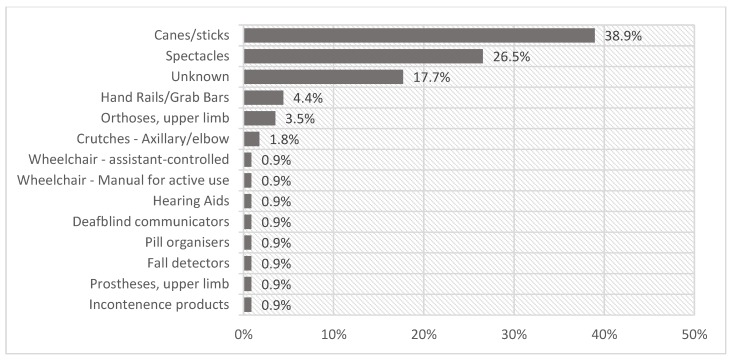
AP used by 112 persons with any difficulty in any domain. Unknown products are where participants identified any ‘other’ device for any domain but no further information was provided.

The proportion of people using AP varied between functional difficulty type (Figure 1). Higher AP use was observed in people with at least some difficulties with self-care (9.9%), walking (9.0%) and seeing (5.1%) compared to people with difficulty remembering (<1%), hearing and communicating (0.5%), and other health problems, not covered in WG questions (0.8%). This figure corresponds to the most frequently reported use of canes/stick (38.9%) and spectacles (26.5%) (Figure 2).

### 3.3. Unmet Needs

The APA-Needs instrument examines two main dimensions of unmet need: (1) the proportion of people who have unmet needs or self-identify a need for AP that they do not already have, and (2) the extent to which current products satisfy needs. First, of people with any functional difficulty, 74.7% reported a need for AP they do not already have. This comprised both 65.3% of people who have any difficulties but no current AP, and 87.5% of people currently using AP. Proportions of respondents reporting unmet need are reported in Table 3. Findings from multivariate analysis using logistic regression predicting self-reported unmet needs for AP (Table 3) suggested that all else being equal, people already using AP were more likely to report an unmet need than people not using AP (OR = 4.95, *p* < 0.01). People who were working were 5.5 times more likely to report unmet need for AP (OR = 5.55, *p* < 0.05) than those who were not working. 

Concerning the self-reported extent to which current devices satisfy needs, when asked ‘does the product meet your needs?’ 75.1% of AP users responded ‘mostly’ or ‘yes’, indicating that existing products mostly or entirely met self-perceived needs. There was no association between functional difficulty levels and satisfaction with AP. 

### 3.4. Market Conditions: Facilitators and Barriers for Using AP

Among people with functional difficulties, the most common source of AP was homemade, self-made, or made by family members (46%). Other sources were hospitals (17.5%) and local markets (11.6%). Other health facilities (3.76%) such as primary health posts or clinics, and government provision (2.2%) were less frequent. In this sample, nobody had received services from rehabilitation centers, Non-Government Organizations or charities.

Most people paid out-of-pocket for their AP. The median cost for products was ৳150 (USD$1.80). Average costs of products increased with the level of functional difficulties, while the median costs decreased with the level of difficulties. This may imply products for more complex needs were more expensive, but that fewer people with higher needs are able to meet the costs.

**Figure 3:**
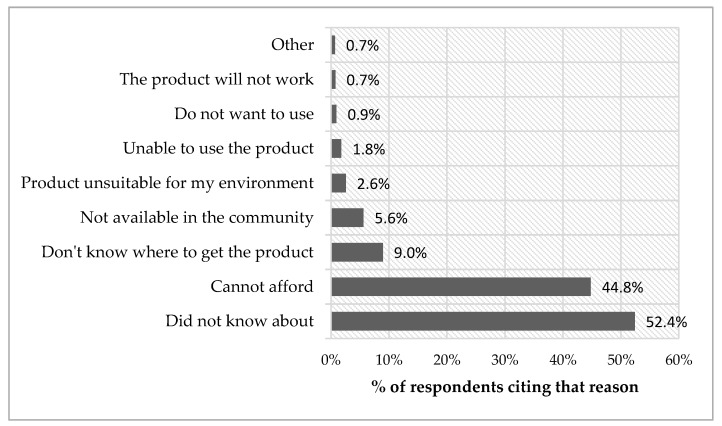
Reasons for not using AP in people with a self-reported unmet need

Affordability (44.8%) and a lack of awareness about AP and where to get them (52.4%) were the most commonly reported reasons for not using an AP in people who self-reported an unmet need (Figure 3). 

## 4. Discussion

Using a new instrument to understand access to and needs for AP, this study estimated 7.1% of adults with any self-reported functional difficulty in the studied population used AP, which was greater than the prevalence of disability. This finding underpins the importance of AP for people other than those who are at risk of disability. It could also indicate for some people, using AP mitigates functional difficulties, and they do not self-identify those difficulties when asked. Among people with any self-reported difficulty, 74.7% had self-reported unmet needs for AP. Use of AP was associated with increasing age and the level of functional difficulties, while access was better in the more urban and wealthy (Narsingdi) of the two studied districts, and people with self-care or walking difficulties. People with a higher level of self-reported functional difficulties, and those who were working, had a higher unmet need for AP. Costs and poor knowledge about options and sources for AP were the most commonly reported reasons for unmet needs. 

Findings are generally consistent with current estimates of unmet need for AP [1,16,17,18,19], however, in this study, overall unmet need (70.7%) is higher than the 14–18% described in a synthesis of studies from higher income countries [20], and from India (56%) [19]. Lower AP use might be associated with the poorer economic conditions of Bangladesh, and the lack of active non-government AP services in the districts we researched. We found a strong positive association between working status (among persons of working age) and self-reported need for AP, suggesting that people who are not working identify less potential benefits of AP than working people.

The high overall unmet need, and the factors associated with unmet need, describe a scenario where sociodemographic characteristics, community knowledge about AP, and cost and supply barriers, prohibit effective coverage and use of AP. Local markets and home ingenuity are the most common solutions. Of those who have access to devices, the relatively high proportion of people who were satisfied (75.1%) implies low-cost products might be suitable for many. However, consistent with recent findings from Kenya [21], people with more difficulties have a greater need. In our study, less service and product availability for more complex products—and affordability—combined to restrict access to AP for people with higher needs compared with others. In the more urban and wealthy Narsingdi, people were more likely to use AP and less likely to express unmet needs, which strengthens assumptions about associations between availability, affordability and appropriateness of AP. 

While our study did not examine whether the devices provided were clinically appropriate, local provision channels are mostly limited to low-cost, simple AP, and are unlikely to involve reasonable clinical assessment and personalized fitting. In Bangladesh, specialized services are not usually available outside major urban cities, except for a small subset of AP—often mobility and vision services. To address these gaps, support from non-government organisations and community-led services might be necessary. However, these options can be costly and complex to sustain [22] and should be a stopgap measure, where local permanent solutions are nascent or unstable.

The finding that most AP is home or self-made also points to modest progress on systems for AP service provision. Despite the poor overall access, unmet need was lower than reported estimates of 85–95% [1,7]. The overall estimate of unmet needs obscures important differences between types and level of difficulty, age and sociodemographic factors. The higher self-reported unmet need in people who currently have at least one AP, might be associated with better knowledge about the potential benefits of assistive products, combined with frustration about the performance of low-cost products. A potential solution to address poor knowledge about options might be to harness local Community Based Rehabilitation (CBR) to provide expertise and accompany consumer-led choices about AP procurement. To address poor access to more complex products, urgent strategies for expanding product range are needed, in balance with minimum standards for provision.

The APA-Needs tool provides a potentially rich, comprehensive and highly practice-relevant dataset. While we used a considerable sample size, it is difficult to make inferences about the availability and use of lower-prevalence products. While we found a lower disability prevalence than estimated, we had meaningful sample size for the main analyses required to address our aims. Further, data were weighted using actual (not assumed) prevalence and sample design effect arising from the complexity of the survey was controlled using appropriate STATA commands. Nonetheless, future studies could use oversampling strategies for AP users and persons with functional difficulties who do not use AP to provide a fuller understanding of current AP use. Furthermore, surveying and analyzing AP suppliers would provide important information to shape interventions for improving coverage and effectiveness of AP. 

A self-reported instrument offers a relatively simple means to understand AP needs and provision, and to give voice to consumer choices about AP, but has important limitations. Self-reporting is likely to produce both under and over-reporting of need. Under-reporting might arise from a lack of knowledge about AP, or low expectations of its value. Self-reporting is known to under-detect participation restrictions and clinical impairments [23], which risks under-reporting of both AP need and use unless AP use questions are asked of all respondents, such as in in the Washington Group Extended Question Set on Functioning (WG ES-F) [24]. Over-reporting of need is likely to arise where other measures are possible, such as a person with cataracts or surgically correctable musculoskeletal impairment, who might benefit from simple and effective surgeries, but who might identify a need for spectacles or mobility products. More work is needed to understand the precision and utility of the ATA-Needs tool and alternative methods for understanding population need and unmet need for AP.

This component of our study did not examine the needs of children, for whom AP are especially important. While our findings are largely consistent with others, access and provision patterns are likely to be highly context-dependent, and caution is needed in generalizing results to other settings. Future research should explore supply-side barriers to addressing more of the needs at affordable costs, and how supply chains and clinical expertise can combine to ensure more needs are met at the same time as improving prescription and adaptation of commercially-supplied products. 

## 5. Conclusions

The combined effects of costs, poor knowledge and availability of products result in profound unmet need, which is a barrier to quality of life improvement and equitable development. Current AP services are not achieving adequate coverage. New strategies should address poor knowledge about AP, while recognizing and building on current supply pathways.

Applying the APA-Needs tool in Bangladesh revealed new insights into provision patterns, with implications for new solutions to improve AP coverage in local communities. These findings have the potential to help address unmet needs for AP, but require commitment, local action and new investments to catalyze change. Our estimates of unmet need highlight the scale of the challenge. Evidence-based responses will require understanding of risk factors for exclusion from AP service provision, predictors of need, and investigations about what solutions are the most effective. These results and the APA-Needs tool thus advance the goal of addressing gaps in access to AP. There is new emphasis on AP as a precursor to disability rights, rehabilitation in health systems and on the potential impact of AP on the lives of their users. The APA-Needs tool’s development is therefore timely.

## Figures and Tables

**Table 1:** Characteristics of assistive products (AP) users and non-users.

Characteristics	AP Users			AP Non-Users			All (User + Non-User)
	Difficulty	No Difficulty	All AP Users	Difficulty	No Difficulty	All AP Non-Users	
Level of difficulties							
None			44.6%			70.9%	69.0%
Some			37.6%			26.2%	27.1%
A lot			11.1%			2.1%	2.7%
Cannot do			6.7%			0.8%	1.2%
Age							
Mean 95% CI	58.1 (55.2–61.0)	41.1 (38.4–43.9)	50.5 (48.1–53.0)	46.2 (45.2–47.3)	33.1 (32.5–33.7)	36.9 (36.4–37.5)	37.9 (37.3–38.5)
Sex							
Female	61.3%	60.3%	60.9%	64.2%	56.8%	59.0%	59.1%
Male	38.7%	39.7%	39.1%	35.8%	43.2%	41.0%	40.9%
School attendance							
Never attended	43.4%	11.3%	29.1%	57.6%	26.8%	35.7%	35.3%
Ever attended	56.6%	88.7%	70.9%	42.4%	73.2%	64.3%	64.7%
Working status							
Not working	80.6%	66.4%	74.3%	69.0%	64.2%	65.6%	66.2%
Working	19.4%	33.6%	25.7%	31.0%	35.8%	34.4%	33.8%
District							
Kurigram	46.0%	34.7%	40.9%	61.1%	48.0%	51.8%	51.0%
Narsingdi	54.0%	65.3%	59.1%	38.9%	52.0%	48.2%	49.0%
Total	100%	100%	100%	100%	100%	100%	100%
*N*	97,629	78,710	176,339	671,903	1,635,387	2,307,290	2,483,629
*n*	189	144	333	1167	2753	3920	4253

Note: *n* is the sample size that was used for analysis; *N* is the population size (weighted and adjusted for complex survey design). ‘Difficulty’ is any difficulty: ‘some’, ‘a lot’, or ‘cannot do at all’.).

**Table 2:** Factors associated with the use of assistive products.

Characteristics	Odds Ratio		95% CI
Level of difficulties			
None (ref.)	1		
Some	2.11	**	1.59–2.83
A lot	6.73	**	3.83–11.86
Cannot do at all	11.03	**	5.67–21.44
Age			
Youth (18–24 years-old) (ref.)	1		
Older adults (25–59 years-old)	2.74	**	1.71–4.41
Elderly (60+ years-old)	7.52	**	4.18–13.55
Sex			
Female (ref.)	1		
Male	1.00		0.68–1.48
School attendance			
Never attended (ref.)	1		
Ever attended	2.92	**	2.08–4.10
Working status			
Not working (ref.)	1		
Working	0.71		0.47–1.07
District of residence			
Kurigram (ref.)	1		
Narsingdi	1.47	*	1.08–2.00
Constant	0.01	**	0.00–0.01
*N*	2,483,629		
*n*	4253		

Note: Logistic regression was used to predict the use of assistive product: 1 = Using an assistive product; 0 = Not using an assistive product; * *p* < 0.05; ** *p* < 0.01. ref. = reference group.

**Table 3:** Unadjusted percentage of people reporting unmet needs and factors associated with self-reported unmet needs.

Characteristics	% Reporting Unmet Needs	OR	95% CI
Level of difficulties			
Some	67.9%	1	0.71–7.05
A lot	83.4%	2.24	0.20–2.64
Cannot do	60.2%	0.73	
Currently using AP			
No	68.2%	1	1.52–15.30
Yes	88.3%	4.82 **	
Age group			
Youth (18–24 years-old)	77.6%	1	0.22–1.96
Older adults (25–59 years-old)	67.1%	0.65	0.32–3.42
Elderly (60+ years-old)	80.7%	1.04	
Sex			
Female	74.9%	1	0.20–1.31
Male	74.4%	0.51	
School attendance			
Never attended	76.6%	1	0.30–1.80
Ever attended	71.1%	0.73	
Working status			
Not working	71.4%	1	
Working	86.2%	5.71 *	1.30–25.16
District of residence			
Kurigram	85.0%	1	
Narsingdi	59.4%	0.34 *	0.13–0.89
All	74.7%		
Constant		9.32	0.95–91.41
*N*	96,815		
*n*	180		

Note: ** Significant difference at *p* < 0.01; * Significant difference at *p* < 0.10.

## Appendix A

**Table A1:** Summary of WHO APA-Needs questions and skip logic as used adapted for use in the current survey.

Level	Section	Questions		Skip Pattern
LEVEL 1—(short version) Need and unmet need	A—Personal details	1–10	Sociodemographic characteristics	
	B—Met and unmet need			
	WG-extended set (example here for walking or climbing steps)	11	In the last 6 months, have you had difficulties WALKING or CLIMBING STEPS?0—no difficulty; 1—some difficulty; 2—a lot of difficulty; 3—cannot do at all	If “0”, go to next function Q; If“2–4”, go to Q12
		12	Do you use any assistive products to: support you in standing, sitting or lyingor move around inside or outside the house or manage other mobility difficulties	If YES, go to Q13; If NO, go to Q15
		13	Which ones do you use?	Go to Q14
		14	Do you need any other products to assist you in these activities? For example: [show corresponding card/s]	If YES, go to Q16; If NO, go to Q17
		15	Do you think you might benefit from any assistive products to assist you in these activities? For example: [show corresponding card/s]	If YES, go to Q16; If NO, go to Q17
		16	Which ones do you need?	Go to Q17
LEVEL 2—(Full version)—Product procurement, performance, use, services, impact; abandonment, barriers	C—AP in use (Repeat for all products reported as used in section B)			
	Procurement	48	Where did you get the [name of product/s from section B]?	
		49	Approximately how much did you pay for [the product]?	
		50	How far did you have to travel to get [the product]?	
		51	How long have you been using the assistive product?	
		52	If the device was lost or broken, are you confident you could easily replace it?	
		53	When you first got [the product] was it new or had it already been used (second-hand)?	
	Performance	54	Does [the product] meet your needs? (does it do everything that you want it to do?)	
		55	In what condition is [the product]?	
		56	What kind of problems have you had with the assistive product?	
		57	In which way do you think [the product] could be better? (what, if anything would you like to change about it?)	
	Use	58	In the typical day, how many hours do you use [the product]?	
		59	How easy is it for you to use [the product] within your home?	
		60	How easy is it for you to use [the product] outside home?	
		61	Do you need any assistance to set up [the product] before using it?	
		62	After set-up, do you need any assistance to use [the product]?	
	Services	63	Was [the product] prescribed for you following an assessment (i.e. looking at you/asking you questions/taking measures)? If yes, by whom?	
		64	Was [the product] fitted to you or customized to your needs? If yes, by whom?	
		65	Have you received any training on how to use and take care of [the product]? If yes, from whom?	
		66	If there is something wrong with [the product] is there somewhere you can go for repairs or maintenance?	
	Impact	67	How important is [the product] for you to do your work or go to school?	
		68	How important is [the product] for you to participate in social events and be an active member of your community?	
		69	How important is [the product] to your health?	
		70	How important is [the product] to your enjoyment or life?	
	Section D—Abandonment & Barriers			
	Abandonment	71	Have you used an assistive product like [the product] in the past?	YES→ Q72; NO→Q76
		72	Do you have an assistive product like [the product] that you no longer use?	YES→ Q73; NO→Q76
		73	How long have you had [product no longer in use]?	
		74	Is [product no longer in use] in working condition?	YES→Q75; NO→Q76
		75	Why are you no longer using [product no longer in use]?	
	Barriers	76	You told me that you do not use assistive products that could help you. What stops you from using them?	
